# *Giardia intestinalis* trophozoites activate human PMN and induce NET formation but dampen neutrophil ROS production

**DOI:** 10.3389/fimmu.2026.1724948

**Published:** 2026-02-20

**Authors:** Constanza Salinas-Varas, Taynar L. Bezerra, Lisbeth Rojas-Barón, Luis F. P. Gondim, Florian Wagenlehner, Ulrich Gärtner, Anja Taubert, Carlos Hermosilla, Iván Conejeros

**Affiliations:** 1 Faculty of Veterinary Medicine, Institute of Parasitology, Justus Liebig University Giessen, Giessen, Germany; 2 Department of Anatomy, Pathology and Clinics, School of Veterinary Medicine and Animal Science, Federal University of Bahia, Salvador, Bahia, Brazil; 3 Clinic of Urology, Pediatric Urology and Andrology, Justus Liebig University Giessen, Giessen, Germany; 4 Institute of Anatomy and Cell Biology, Justus Liebig University Giessen, Giessen, Germany

**Keywords:** ESPs, *Giardia intestinalis*, Giardiasis, NET, PMN, ROS

## Abstract

**Introduction:**

*Giardia intestinalis* is a zoonotic enteric protozoan parasite causing giardiasis inhumans, domestic animals and wildlife. More than 300 million human cases of diarrhea due to giardiasis have annually been reported. Despite its high global prevalence, human polymorphonuclear neutrophil (PMN)-mediated early innate immune responses against *G. intestinalis* remain poorly investigated. This study aimed to evaluate whether vital *G. intestinalis* trophozoites activate PMN and foster neutrophil metabolic responses, thereby eventually driving NET formation.

**Methods:**

Human PMN were exposed to *G. intestinalis* trophozoites and *Giardia*-derived excretory/secretory products (ESPs); stimulation of PMN with PMA served as positive control for both NET induction and neutrophil oxidative (OCR) and glycolytic (PER) responses.

**Results and discussion:**

NET release was illustrated by scanning electron microscopy (SEM), confirmed and quantified by fluorescence microscopy via the co-localization of histone, neutrophil elastase (NE) and extracellular DNA. PMN activation and metabolic responses were assessed at the level of oxygen consumption rates (OCR), proton efflux rates (PER), and ROS production. Microscopy analyses showed that vital *G. intestinalis* trophozoites activated PMN, triggering neutrophil phagocytosis and NET-based entrapment of trophozoites. Furthermore, the presence of PMN in trophozoite growth cultures dampened parasite replication efficacy. Trophozoite exposure fostered both OCR and glycolytic PER responses in PMN but failed to drive neutrophil ROS production. To investigate whether the lack of ROS production is a *Giardia*-mediated immune evasion strategy, the ability of *G. intestinalis* trophozoites to inhibit PMA-induced ROS generation in neutrophils was assessed. Trophozoites significantly diminished PMA-driven ROS production, impairing a key PMN effector mechanism. To elucidate if these effects were based on parasite-derived products, *Giardia*-ESPs were tested for their effects on neutrophil metabolic responses and PMA-mediated ROS production. No changes were observed, excluding ESPs-driven effects. In conclusion, our results showed that *G. intestinalis* trophozoites activate human PMN on an oxidative- and glycolytic level, stimulating them to extrude NETs and/or to engage in phagocytosis, and to impair parasite’s binary fission.

## 1 Introduction

*Giardia intestinalis* (syn. *Giardia duodenalis*) is a neglected zoonotic enteric protozoan parasite causing giardiasis, a disease affecting a wide range of hosts, including humans, domestic animals and wildlife (1, 2). This global parasitosis causes more than 300 million human cases of diarrhea annually (3). With a prevalence of up to 30% in developing countries (4), *G. intestinalis* is one of the world’s most prevalent pathogenic protozoa (5). Acute *G. intestinalis* infection in humans induce a wide range of clinical signs including catarrhal diarrhea, cramps, nausea, vomiting, and urticaria (6). Additionally, irritable bowel syndrome (IBS) and chronic fatigue have been reported as sequelae of human giardiasis (6, 7). Although a high percentage of human giardiasis cases may remain asymptomatic and self-limited, life-long consequences, including impaired physical and cognitive development have been reported in *G. intestinalis*-infected young children (5, 8).

The direct life cycle of the parasite includes a switch between two morphological different stages, the vegetative and motile multi-flagellated trophozoite form (i. e. parasitic form) and the infective cyst form (9). Exogenous *G. intestinalis* cysts can survive in cool and moist environments for months until infecting a new host via fecal-oral transmission (10). Once ingested, *G. intestinalis* cysts reach the upper intestinal tract, where two trophozoites are released during the excystation process (11). Herein, extracellular trophozoites rapidly multiply asexually and colonize the small intestine mucosa, generating villi damage and local inflammation resulting in malabsorption and diarrhea (7). Eventually, the encystation process of trophozoites starts within the large intestine, and new resilient cysts are shed in host’s feces, ensuring successful transmission (12). Trophozoites typically reside on the surface of intestinal epithelial cells (IEC) of the small intestine (13), nevertheless, the firm adhesion via the ventral adhesion discs to IEC triggers cell stress and damage. Consequently, an immediate innate immune response is mounted (14, 15). Here external pathogen-associated molecular patterns (PAMPs) or body’s own danger-associated molecular patterns (DAMPs) are recognized leading to the release of alarm molecules (e. g. ATP, exRNA, exDNA, calprotectin) (16), which is followed by a local inflammatory response, including the production of chemokines and cytokines (17, 18). The subsequent recruitment and accumulation of circulating polymorphonuclear neutrophils (PMN), monocytes and macrophages in a sequential order to the site of infection culminates in their phagocytic actions to remove cell debris and pathogens (19). As such, *in vivo* histological studies on *G. intestinalis*-infected gut mucosa show significant infiltration by PMN, monocytes and macrophages (20, 21). Later on, also intraepithelial lymphocytes (IEL), dendritic cells (DC) as well as lamina propria lymphocytes (LPL) are recruited to the site of *G. intestinalis* infection (6, 22, 23). Interestingly, human PMN and monocytes were documented to interfere with *G. intestinalis* trophozoite adherence to IEC (24). Both azurophil- as well as specific granule-derived molecules isolated from stimulated PMN and monocytes were equally efficient to block trophozoite adherence (24). Furthermore, PMN-derived ROS also showed anti-giardial activities hampering trophozoite adhesion and proliferation (24). In addition, antimicrobial peptides derived from activated PMN, monocytes and/or IEC also showed anti-trophozoite effects. Thus, Aley et al. (25) showed that defensins and antimicrobial peptides promoted trophozoite surface destruction, thereby decreasing parasite viability. Moreover, the human neutrophil peptide 1 (HNP-1) as well as the rabbit neutrophil peptide 2 (RNP-2) have been demonstrated to hamper *Giardia* trophozoite replication (6, 25). Other anti-giardial peptides are the bovine-derived indolicidin and the intestinal Paneth cell-derived α-defensins cryptdins 2 and cryptdins 3. Both cryptdins significantly decreased trophozoite viability *in vitro* (25). PMN-derived lactoferrin and lactoferricin also affected *Giardia* growth *in vitro* thereby promoting the encystation process by a *Giardia*-specific low-density lipoprotein receptor (GlLPR) (26). Conversely, to evade the host immune defense and ensure a successful colonization, *G. intestinalis* trophozoites secrete diverse proteins during the interaction with host cells, known as excretory/secretory products (ESPs) (11, 27). *Giardia*-derived ESPs are capable to modulate the host cell metabolism and immune responses, as evidenced by the downregulation of immune signaling and promotion cell apoptosis (27–29). The parasite evade PMN defensin-driven effects by the cleavage of human β-defensin 1 (β-HD1) and α-human defensin 6 (α-HD6) via secreted cysteine proteases (30). Moreover, *G. intestinalis*-secreted cathepsin B cysteine proteases can cleave CXCL-8, thereby attenuating PMN chemotaxis and inhibiting PMN recruitment via CXCL8/CXCR1/CXCR2 circuit (28, 31). Despite the close interaction between host- and parasite-derived proteins, the biological role of *G. intestinalis*-derived ESPs on human PMN-neutrophil extracellular traps (NETs) mechanism is yet to be defined.

As already stated, *G. intestinalis* attachment leads to apoptosis and disruption of the IEC tight junction barrier (32). This tissue damage promotes immediate recruitment of PMN into the gut (14). PMN fulfill their antimicrobial role through a wide range of defensive mechanisms, including production of reactive oxygen species (ROS) and pro-inflammatory chemokines/cytokines, phagocytosis, extracellular vesicle (EV)- and NETs release (33, 34). NETs are a network of extracellular strings composed by nuclear and mitochondrial DNA and granular enzymes with microbicidal activity, such as neutrophil elastase (NE), pentraxin, lactoferrin, defensins, LL37 and myeloperoxidase (MPO) (35). The expulsion of these web-like structures facilitates entrapment and neutralization of different invasive pathogens like bacteria, viruses, fungi, and parasites (36–38). Diverse intra- and extracellular parasites foster the release of NETs, including *Eimeria bovis*, *Eimeria arloingi*, *Besnoitia besnoiti*, *Neospora caninum*, *Toxoplasma gondii*, *Cryptosporidium parvum* and *Trypanosoma brucei brucei* (39–46), however, related studies on *G. intestinalis* are scarce. So far, two reports aimed to study *G. intestinalis*-induced NET formation in the bovine (47) and human system (48). In the case of *Giardia*-induced bovine NETosis, neutrophil purinergic signaling seemed of high relevance (47), which is in accordance with apicomplexan and euglenozoan parasite-mediated NETosis (40, 49). However, in the human system, PMN failed to respond by NETs when exposed to *G. intestinalis* trophozoites (48). Therefore, the current work aimed to study the capacity of vital *G. intestinalis* trophozoites and *Giardia*-derived ESPs to activate human PMN, resulting in NETs release. Therefore, we studied human PMN activation after trophozoite exposure at the level of oxygen consumption rates (OCR), proton efflux rates (PER), and ROS production. Scanning electron microscopy (SEM), as well as immunofluorescence microscopy (IFM) analyses unveiled *G. intestinalis*-triggered human NETosis resulting in entrapment of trophozoites. Despite the trophozoite’s size and motility, phagocytic activities of single PMN were also observed. However, if *G. intestinalis*-mediated human NETosis will limit the extent of trophozoite-associated intestinal damage *in vivo* needs further investigation. The same holds true for the involvement of both pathogen-recognition receptors (PRR) and signaling pathways controlling human PMN responses.

## 2 Materials and methods

### 2.1 Ethics statement

All adult blood donors were informed on the study and were recruited on a voluntary basis. The anonymity of the donors was guaranteed at all times. All blood donors signed a written consent form before participating in this study. Human blood sampling was conducted according to ethical vote number AZ: 32/11 assigned to Prof. Dr. Florian Wagenlehner, Head of the Clinic for Urology, Pediatric Urology and Andrology, approved by the Faculty of Medicine’s Institutional Safety Commission of the Justus Liebig University Giessen, Germany, and following the standards set by the Declaration of Helsinki.

### 2.2 *Giardia intestinalis in vitro* culture

Microaerophilic *G. intestinalis* trophozoites (strain WB; ATCC) were axenically cultured in TYI-S-33 complete medium at 37 °C and 5% CO_2_. TYI-S-33 medium was prepared based on Keister et al. (50) with the following composition: 18 g/L casein peptone (#1.07213, Merck), 9.0 g/L yeast extract (#212750, Thermo Fischer), 10 g/L glucose (#49159, Merck), 2.0 g/L NaCl (#3957.1, Roth), 0.2 g/L L-ascorbic acid (#A5960, Sigma-Aldrich), 1.0 g/L K_2_HPO_4_ (#5104, Merck), 0.6 g/L KH_2_PO_4_ (#3904.1, Roth), 4.0 g/L L-cysteine (#30129, Merck), 0.2 g/L ferric ammonium citrate. The medium was supplemented with 10% heat-inactivated newborn calf serum (N4637, Sigma-Aldrich) and 52 mg/mL bovine bile (B3883, Merck). The pH was adjusted to 7.0 and medium filter-sterilized (0.22 µm; 83.3941.511, Sarstedt). Parasites in the growth phase were placed on an ice bath for 20 min to allow the detachment of trophozoites, and harvested by centrifugation at 650 × *g* for 10 min at 4 °C. The parasites were counted and placed in the incubator at 37 °C and a 5% CO_2_ atmosphere until further use.

### 2.3 Isolation of *Giardia intestinalis* excretory/secretory products (ESPs)

Supernatants of confluent *G. intestinalis* were collected and centrifuged for 20 min at 4500 × *g* and 2 °C. Collected supernatants were sterile filtered using a 5.0 µm filter (SLSV025LS, Millex^®^-SV). Then, cell-free supernatant samples were transferred to an Amicon centrifugal filter 100K (UFC910024, Amicon^®^ Ultra-15). Samples were concentrated by centrifugation at 4000 × *g* and 2 °C until 500 µL of supernatant was obtained. Concentrated ESPs samples were quantified using Pierce™ BCA protein assay kit (#23227, Thermo Fischer) according to the manufacturer’s instructions and stored at -80 °C until further use.

### 2.4 Isolation of human PMN

PMN were isolated from freshly collected peripheral blood of adult healthy donors (*n* = 10; 4 males; 6 females). Peripheral blood samples were obtained by venipuncture of the antecubital veins and collected in EDTA sterile tubes (#02.263, Monovette, Sarstedt). PMN were isolated using an EasySep™ Direct Human Neutrophil Isolation Kit (#19666, StemCell Technologies) following the manufacturer’s instructions. PMN were suspended in a modified RPMI 1640 medium without phenol red (#R7509, Sigma-Aldrich), counted, and maintained on ice until further use.

### 2.5 Scanning electron microscopy analysis

PMN (3 ×10^5^; *n* = 3) were co-cultured with vital *G. intestinalis* trophozoites at a 1:3 ratio on 0.001% poly-L-lysine (#P8920, Sigma-Aldrich) pre-coated 10 mm coverslips for 180 min at 37 °C and 5% CO_2_. Cells were fixed in 2.5% glutaraldehyde (Merck), post-fixed in 1% osmium tetroxide (Merck), and gently washed in distilled water. The process was followed by dehydration of the samples, critical point drying by CO_2_ treatment, and sputtering with gold. Finally, all samples were analyzed via a Philips XL30 scanning electron microscope (Institute of Anatomy and Cell Biology, JLU Giessen, Germany).

### 2.6 NET detection by immunofluorescence microscopy

PMN (3 ×10^5^; *n* = 3) were confronted with vital *G. intestinalis* trophozoites at a 1:3 ratio, ESPs (5.0 µg/well), phorbol-12-myristat-13-acetat PMA (PMA; 100 nM; #P1585, Sigma-Aldrich) or a combination of two of the stimuli to evaluate the effects of *G. intestinalis* trophozoites or ESPs on PMA-activated PMN. Samples were incubated for 180 min at 37 °C and 5% CO_2_ in a 24-well plate (#353047, Greiner) containing fibronectin (#F1141, Sigma-Aldrich) pre-coated 10 mm coverslips. As controls, unstimulated PMN and PMA-activated PMN were used. After incubation, cells were fixed with 4% (w/v) paraformaldehyde (# 4979.1, Roth) for 15 min at room temperature (RT) and washed thrice with sterile PBS (pH 7.4). Samples were then incubated in blocking/permeabilization buffer [PBS; 3% bovine serum albumin (BSA); 0.3%, Triton X-100; all Sigma-Aldrich] for 1h at RT. For the detection of NE and pan-histone within the NET structures, the samples were reacted overnight at 4 °C with polyclonal anti-NE antibodies (1:200, rabbit, #Ab68672, Abcam) and anti-histone clone H11–4 monoclonal antibodies (1:200; mouse, #MAB3422, Millipore). Thereafter, the samples were gently washed three times with sterile PBS and incubated for 30 min in secondary antibody solutions (Alexa fluor 488-conjugated goat anti-rabbit IgG, 1:500; A11008, Invitrogen, or Alexa fluor 594-conjugated goat anti-mouse IgG, 1:500; A11005, Invitrogen; RT, protected from light). Finally, the samples were washed in sterile PBS and mounted with antifading DAPI-Fluoromount-G^®^ medium (#0100-20, Southern Biotech). Images were acquired via a ReScan^®^ confocal instrumentation (RCM 1.1 Visible, Confocal.nl) combined with a Nikon Eclipse Ti2-A inverted microscope. Identical brightness and contrast conditions were applied for each data set within one experiment. Images were processed using Image J Fiji version software (ImageJ 1.54f, USA). Events positive for histone signal were divided by the total number of DAPI-positive cells and multiplied by 100 to calculate the percentage of NET-forming cells.

### 2.7 Quantification of reactive oxygen species (ROS)

ROS was measured by a chemiluminescence-based assay using luminol (#A4685, Sigma-Aldrich). Briefly, 5 × 10^5^ PMN (*n* = 4-9) suspended in modified RPMI 1640 medium without phenol red (#R7509, Sigma-Aldrich) were seeded per well in a 96-well white flat bottom plate (#655074, Greiner). Stock luminol solution was prepared at 50 mM concentration diluted in 0.1 M NaOH, and added to each well at a final concentration of 7.5 µM. Baseline luminescence measurements of 20 readings without stimulus were obtained at the beginning of the experiment, followed by PMN stimulation with either *G. intestinalis* trophozoites at different ratios (1.5 × 10^6^; 1.5 × 10^7^), PMA (100 nM; #P1585, Sigma-Aldrich), ESPs (1.0 µg/well and 5.0 µg/well), or a combination of two stimuli to evaluate effects of *G. intestinalis* trophozoites and parasite-ESPs on PMA-induced ROS production. For controls, *G. intestinalis* trophozoites, unstimulated PMN and PMN in an equivalent volume of complete TYI-S-33 medium were used. All experiments were assessed in duplicates, and results were expressed as relative luminescence units (RLU). Chemiluminescence was measured for 250 min in a luminometer (Luminoskan, Thermo Scientific).

### 2.8 Assessment of oxygen consumption rates and proton efflux rates in human PMN stimulated with *G. intestinalis* trophozoite and *Giardia*-derived ESPs

The metabolic activation of PMN exposed to *G. intestinalis* trophozoites was assessed by the measurement of OCR and PER in a Seahorse XFe96^®^ analyzer (Seahorse Bioscience, Agilent Technologies, USA). PMN (*n* = 6-9) were suspended in freshly prepared Seahorse XF RPMI assay medium (pH 7.4, #103576-100, Agilent), supplemented with 10 mM glucose (#103577-100, Agilent), 1 mM pyruvate (#103578-100, Agilent) and 2 mM L-glutamine (#103579-100, Agilent). PMN were seeded at a density of 2.0 ×10^5^ cells per well in an XF96 cell culture plate (Agilent) pre-coated with fibronectin (#F1141, Sigma-Aldrich) and centrifuged (1 min, 200 × *g*, without brake, RT) to facilitate their adherence to the bottom of the cell culture plate. PMN were then allowed to equilibrate for 30 min at 37 °C without CO_2_ supplementation. Thereafter, pre-warmed (37 °C) XF RPMI assay medium (Agilent) was added to each well to a final volume of 180 µl, and the plate was returned to the non-CO_2_-perfused incubator for further 20 min. *G. intestinalis* trophozoites were suspended at a density of 6 ×10^5^ or 6 ×10^6^ parasites/20 µl TYI-S-33 complete medium and placed in one of the four injection ports of the sensor cartridge (Agilent), which was pre-hydrated for 24 h prior to the assay, with calibrant solution (Agilent) in a non-CO_2_-perfused incubator at 37 °C. The effect of *Giardia*-derived ESPs on PMN-OCR and -PER values was also studied. ESPs were placed in one of the four ports of the sensor cartridge at a final concentration of 1.0 µg/well and 5.0 µg/well. For all experiments, controls injection ports were loaded with either XF RPMI assay medium (Agilent), complete TYI-S-33 medium or 100 nM PMA (#P1585, Sigma-Aldrich). The protocol included three basal measurements followed by the injection of medium, vital *G. intestinalis* trophozoites (MOI, PMN to *G. intestinalis* trophozoites, 1:3 and 1:30), ESPs (1.0 µg/well and 5.0 µg/well) and/or PMA followed by a total measurement period of 200 min. Background subtraction, determination of OCR, and conversion of extracellular acidification rate (ECAR) to PER values were performed using the online available Agilent Seahorse Analytics software (https://seahorseanalytics.agilent.com). Area under the curve (AUC) analyses were performed using GraphPad Prism software (GraphPad Software Inc., V10.4.1; La Jolla, CA, USA).

### 2.9 Effects of human PMN presence on *G. intestinalis* binary fission rate

To determine if *G. intestinalis* trophozoite binary fission (i. e. asexual reproduction) was affected by exposure to PMN, PMN (1 × 10^6^; *n* = 4), suspended in modified RPMI 1640 medium without phenol red (#R7509, Sigma-Aldrich) were co-cultured with vital *G. intestinalis* trophozoites suspended in complete TYI-S-33 medium at a 1:3 ratio. TYI-S-medium account for only 8.5% of the final experimental volume, and the same amount of TY-I-S-33 medium was added to control conditions. The cells were incubated at 37 °C and 5% CO_2_ in a 24-well plate (#353047, Greiner). Trophozoite asexual reproduction and viability were assessed by direct cell counting after trypan blue staining (3 min, #93595, Sigma Aldrich). Cells permeable for trypan blue were identified as dead and excluded from further quantification. Cell counting was performed in a Neubauer chamber (Blaubrand, Germany) at 1, 2, 4, 18, 24 and 48 h post seeding.

### 2.10 Statistical analysis

For ROS production and Seahorse-related experiments, AUC values were calculated and used for statistical analysis. Normality of the data was assessed via Shapiro-Wilk test. Seahorse-PER values, IFA quantification, and ESPs-derived ROS production values were analysed by one way analysis of variance (ANOVA) followed by Tukey´s multiple comparisons test. Seahorse-OCR *p*-values were determined by Kruskal-Wallis test followed by Dunn´s multiple comparisons test. For parasite replication assessment, an unpaired two-tailed t-test was conducted for each time point. Additionally, a non-linear regression analysis fitted to an exponential growth model was performed. For each experiment, the number of biological replicates is specified in the Material and Methods section. All graphs and statistical analyses were performed using GraphPad Prism^®^ software (GraphPad Software Inc., V10.4.1; La Jolla, CA, USA). Data is presented as mean ± standard deviation (SD), represented by error bars in all figures. Statistical significance was defined as *p*-value ≤ 0.05.

## 3 Results

### 3.1 *G. intestinalis* trophozoites and *Giardia*-derived ESPs trigger low incidence NET release by human PMN

**Figure 1:**
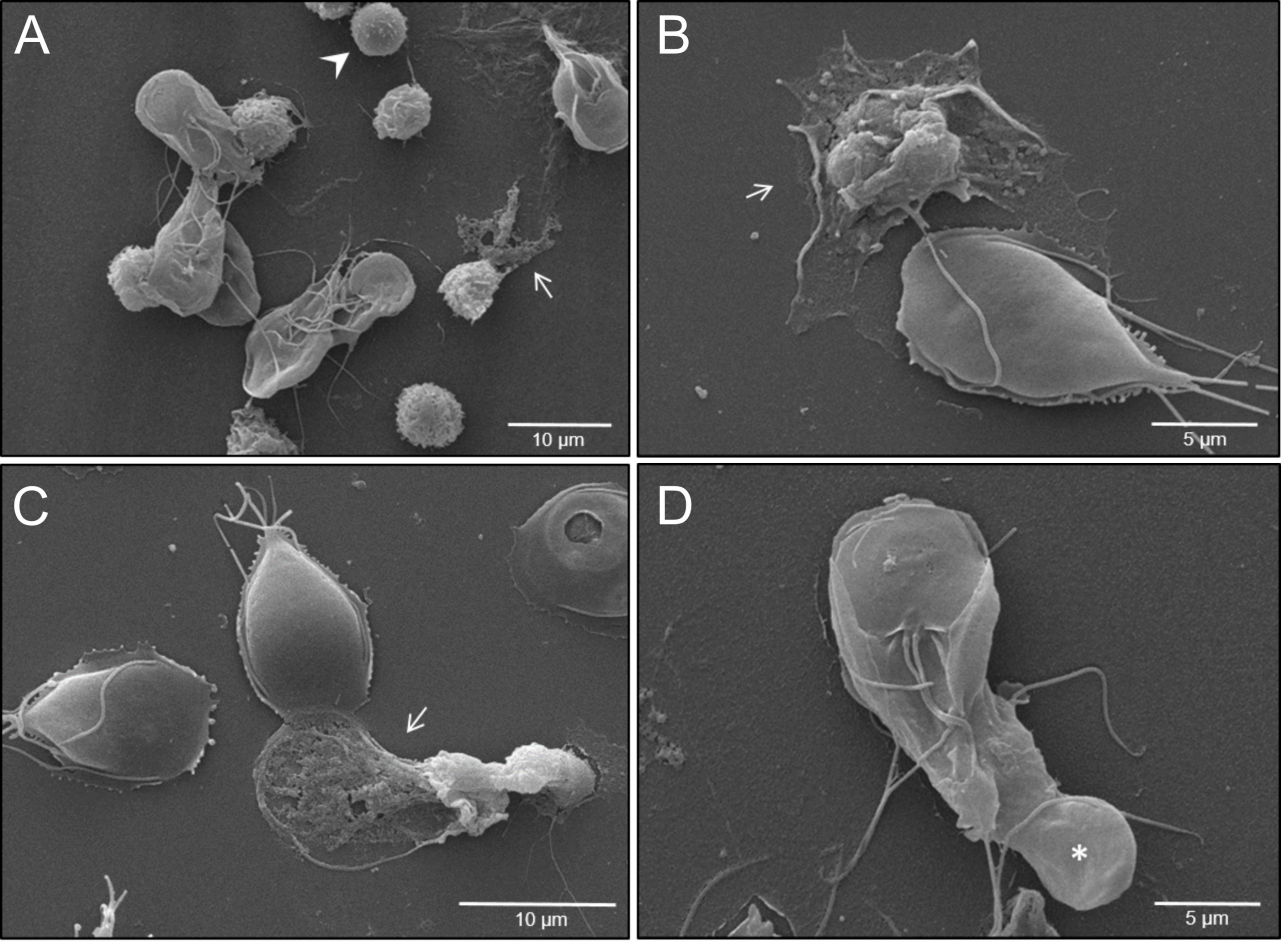
*Giardia intestinalis* trophozoites trigger PMN activation, NET formation and phagocytosis in human PMN. **(A)** PMN-parasite interactions were studied by scanning electron microscopy (SEM). Activated PMN (rough surface) as well as PMN releasing NETs (white arrows) and non-activated PMN (white arrowhead) can be observed in PMN-trophozoite-co-cultures. **(B, C)** Note released NETs (white arrows) and trophozoites exposing their dorsal surface. **(D)** Attempt of phagocytosis of *G. intestinalis* trophozoite from a single PMN (white asterisk). The ventral adhesion disc and multiple flagella of a single *G. intestinalis* trophozoite can be observed.

**Figure 2:**
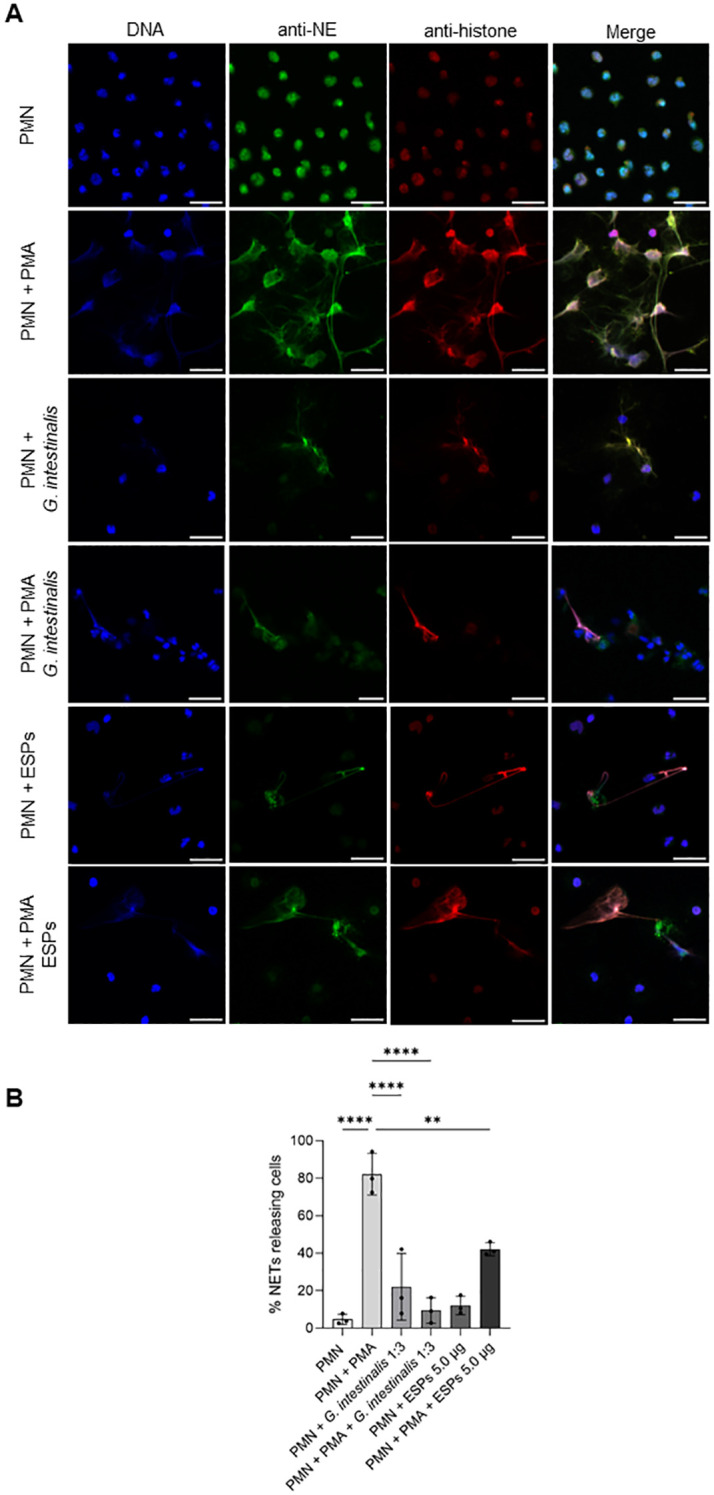
*Giardia intestinalis* trophozoites and *Giardia*-derived excretory/secretory products (ESPs) trigger low incidence NET release in human PMN. **(A)** PMN were cultured without stimuli (negative control), in the presence of vital *G. intestinalis* trophozoites (MOI 1:3), ESPs, PMA or a combination of both stimuli for 180 min. Immunofluorescence microscopy revealed NET structures by typical extracellularly released structures showing co-localization of decondensated DNA (DAPI, blue), neutrophil elastase (NE, green) and pan-histone (red). **(B)** Quantification of PMN-NET formation after the exposure to different stimuli. Images are representative of three independent experiments. Scale bars = 20 µm. **p ≤ 0.01; ****p ≤ 0.0001.

**Figure 3:**
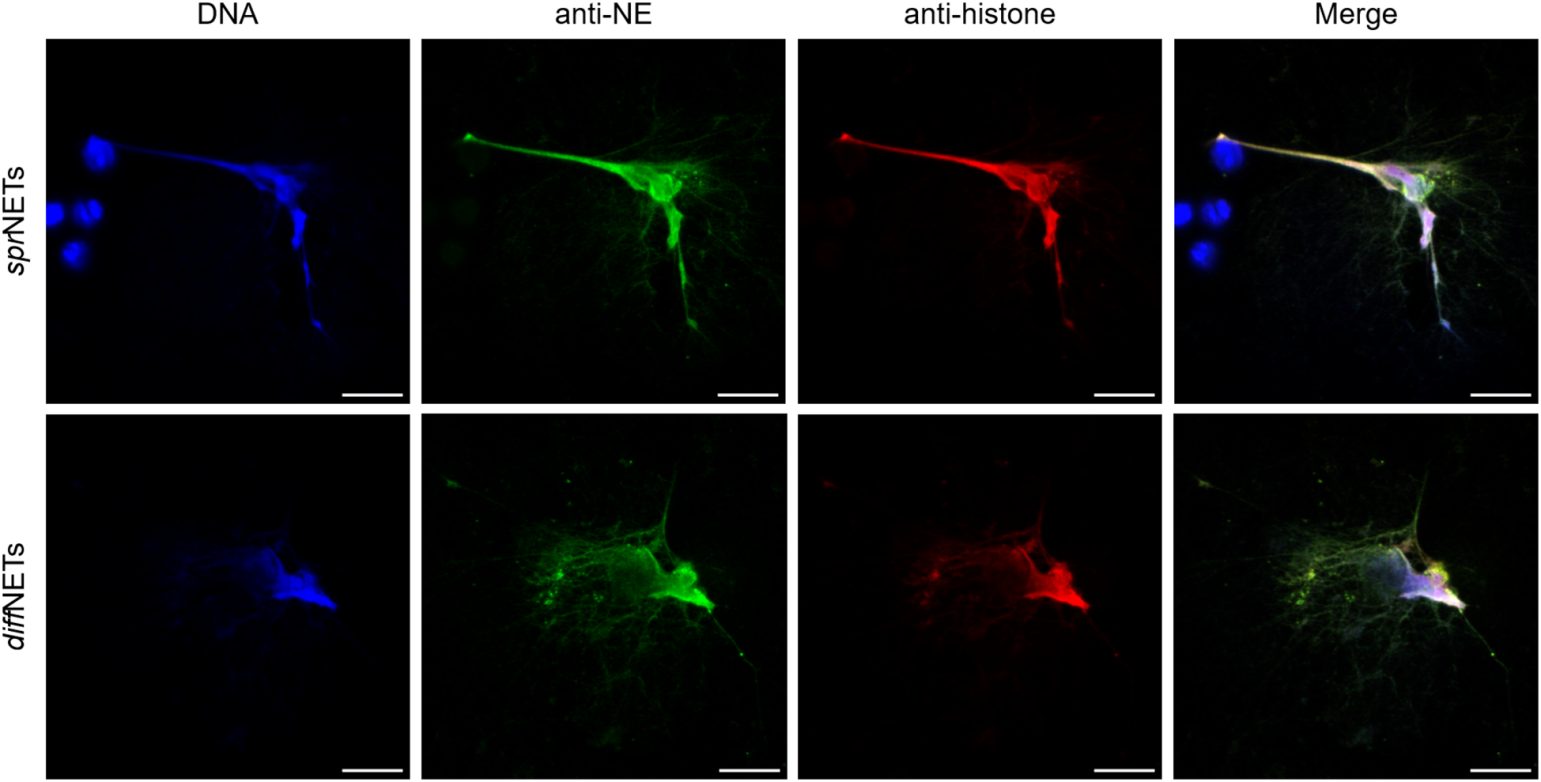
*Giardia intestinalis* trophozoites induce the release of different NET phenotypes by human PMN. A spread NET (*spr*NETs) phenotype is exemplarily illustrated in the upper row. Note the thin and long strings of extracellular DNA (blue) co-localizing with neutrophil elastase (NE; green), and pan-histone (red). Diffuse NETs (*diff*NETs) are exemplarily illustrated in the lower row and consist of roundish structures of decondensated DNA and effector molecules, as demonstrated by the co-localization of DNA (blue), NE (green) and pan-histone (red). Scale bars = 10 µm.

SEM analysis illustrated that exposure of PMN to vital *G. intestinalis* trophozoites activated PMN and, in principle, triggered the release of NET-like structures, even though at low incidence (**Figure 1**). SEM images reveal diverse PMN-parasite-interactions, including different phases of PMN activation ranging from round, smooth surfaced non-activated cells (**Figure 1A**, arrowhead) to activated PMN with irregular shapes and rougher surfaces (**Figures 1A**–**C**, white arrows). Additionally, and despite the size and motility of *G. intestinalis* trophozoites, an attempt of phagocytosis by a single PMN is illustrated, resulting in partial parasite engulfment (**Figure 1D**). Classical characteristics of NETs were confirmed via immunofluorescence assays, by co-localization of pan-histone and NE with DNA-positive extracellular structures (**Figure 2A**). As expected, a significant increase in the percentage of NET releasing cells was observed for PMA-activated PMN, when compared to unstimulated cells (*p* < 0.0001) (**Figure 2B**). Although *G. intestinalis* stimulate PMN to release NET in a low incidence manner, no significant differences were observed in relation to unstimulated PMN (*p* = 0.286) (**Figure 2B**). Interestingly, PMA-activated cells in the presence of *G. intestinalis* significantly reduced (*p* < 0.0001) NET release, when compared to PMA-activated cells (**Figure 2B**). On the other hand, *Giardia*-derived ESPs failed to trigger the release of NET by PMN (*p* = 0.923) (**Figure 2B**). However, when studied the effect of ESPs on PMA-activated PMN, a significant reduction in NET was observed (*p* = 0.002) when compared to PMA-activated cells (**Figure 2B**). NETs phenotypes were also evaluated (**Figure 3**). NETs can be classified based on their phenotypes into spread NETs (*spr*NETs), diffuse NETs (*diff*NETs), or aggregated NETs (*agg*NETs) (51–53); *diff*NETs, showing a more circular web-like structure (**Figure 3**, lower row), and elongated *spr*NETs, represented by fine lines of extracellular DNA, NE and pan-histone (**Figure 3, upper row**), were detected in response to vital *Giardia*-trophozoites exposure.

### 3.2 *G. intestinalis* trophozoite exposure boosts oxidative- and glycolytic responses in human PMN

**Figure 4:**
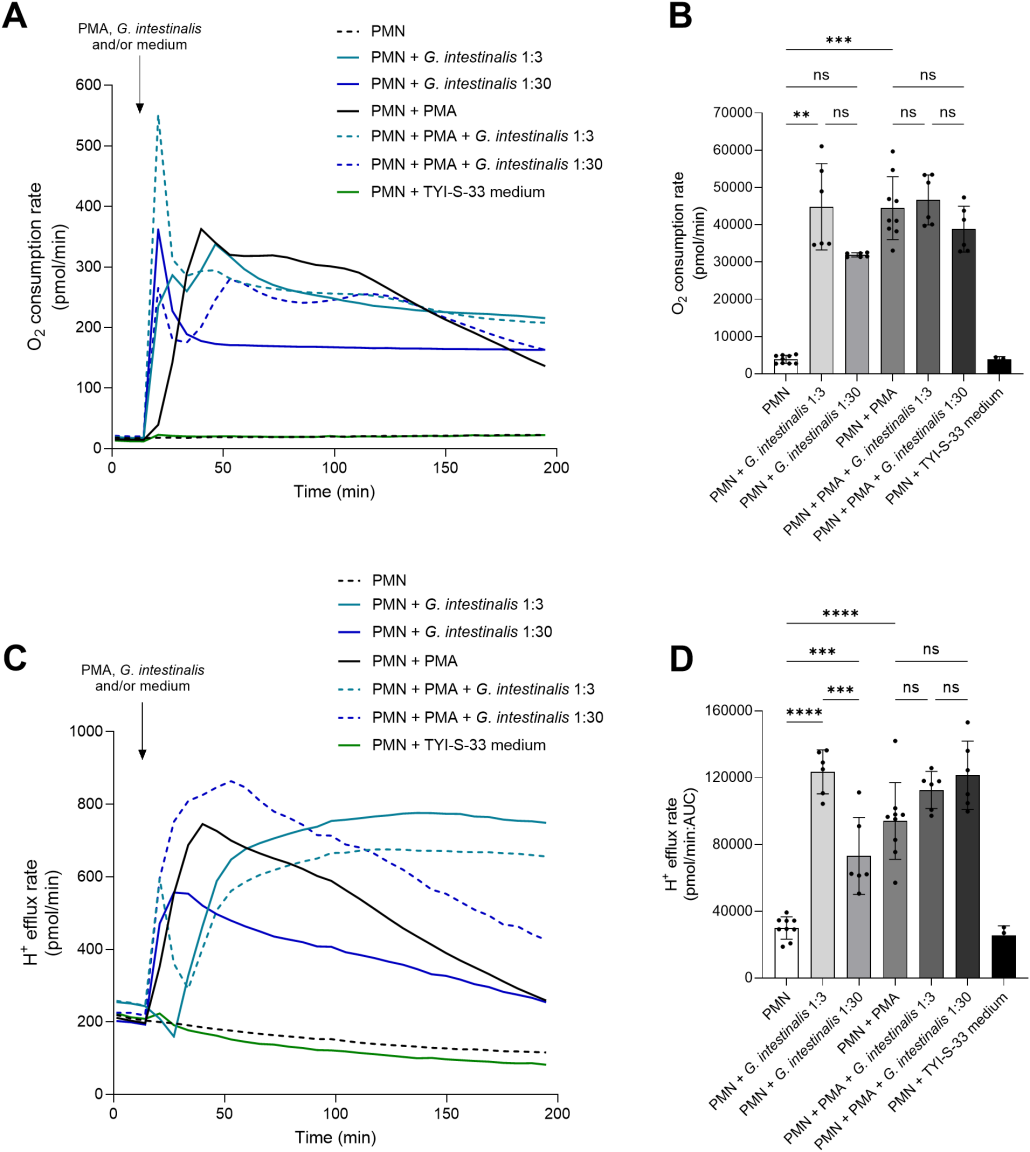
*Giardia intestinalis* trophozoites drive oxidative and glycolytic responses in human PMN. **(A, C)** The bioenergetic status of parasite-exposed PMN was measured on the level of oxygen consumption (OCR) and proton efflux (PER) rates using a Seahorse XFe96 extracellular flux analyzer over a period of 200 min **(B, D)**. The area under de curve (AUC) was calculated for all registries and plotted as mean ± SD. A significant increase in the oxidative (OCR) and glycolytic (PER) activity of parasite-exposed PMN were observed at a 1:3 ratio and in PMA-activated cells. **p ≤ 0.01; ***p ≤ 0.001; ****p ≤ 0.0001; ns, not significant.

To analyze whether parasite encounter leads to granulocytic metabolic responses reflecting PMN activation, real-time changes in the oxidative (OCR) and glycolytic (PER) metabolism of PMN confronted with a low (MOI: 1:3) and high number (MOI: 1:30) of *G. intestinalis* trophozoites were studied (**Figure 4**). After setting baselines in unstimulated PMN, cells were exposed to plain medium, motile *G. intestinalis* trophozoites, PMA, or a combination of both. *Giardia* trophozoites confrontation induced oxidative responses in PMN by upregulating OCR at 1:3 MOI (*p* = 0.003) (**Figures 4A, B**). Furthermore, *G. intestinalis* was able to induce glycolytic metabolic changes (PER) at both ratios (MOI: 1:3, *p* < 0.0001; MOI: 1:30, *p* = 0.0004) (**Figures 4C, D**). Interestingly, the higher MOI (1:30) induced lower oxidative- and glycolytic responses than lower parasites numbers (1:3 vs 1:30), although with significant differences observed only for PER (*p* = 0.0002) (**Figure 4**). Moreover, given that *Giardia* trophozoites have been shown to manipulate epithelial cell reactivity (14), we tested whether the parasite presence would affect PMA-driven responses using two different MOIs (1:3 and 1:30). As expected, an increase in OCR (*p* = 0.0005) and PER (*p* < 0.0001) values were detected in PMA-stimulated PMN, with no differences observed for parasite-PMA-driven OCR and PER responses, independently of the MOI studied (**Figure 4**).

### 3.3 Presence of *G. intestinalis* trophozoites dampens PMA-driven ROS production in human PMN

**Figure 5:**
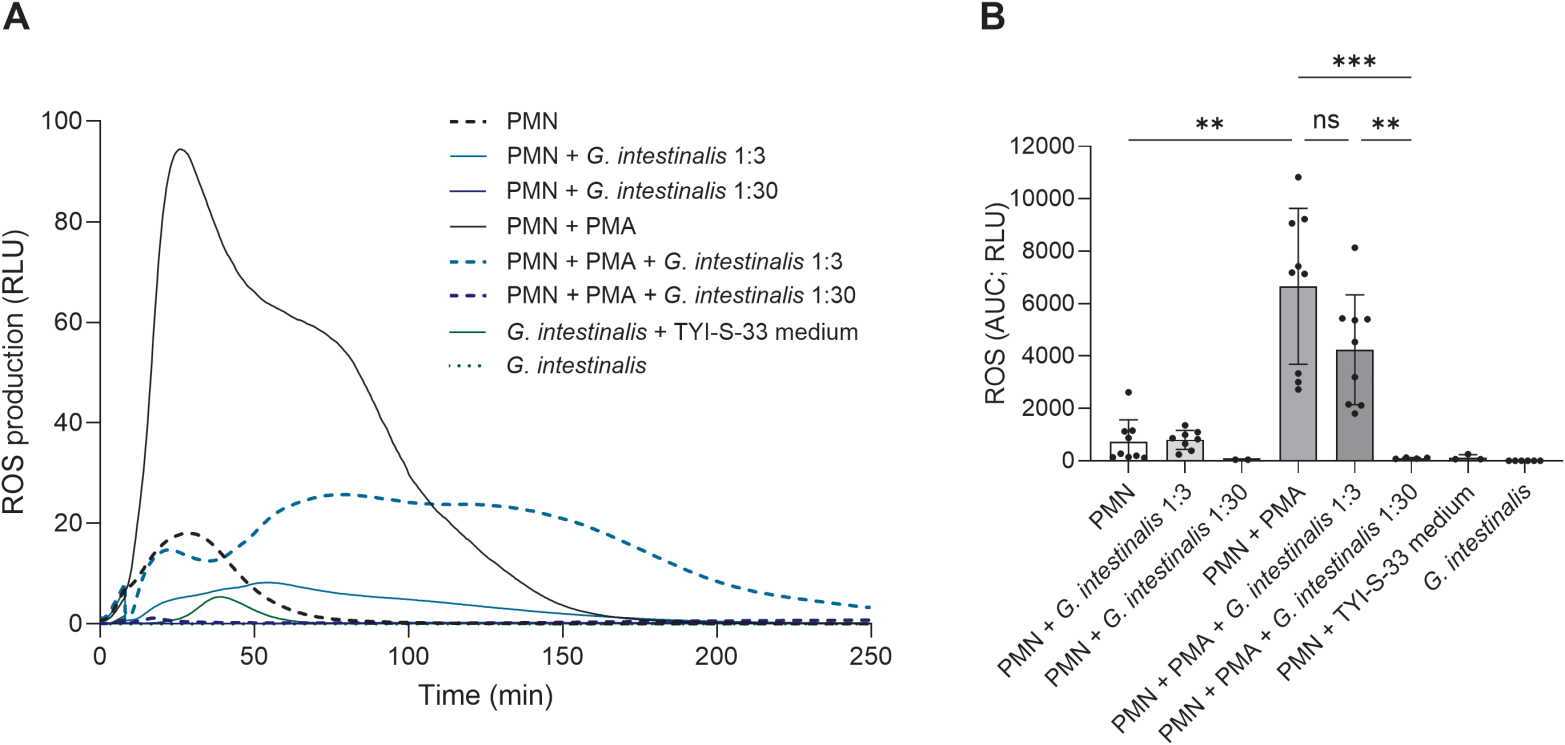
*Giardia intestinalis* trophozoites fail to trigger reactive oxygen species (ROS) production and dampen PMA-induced oxidative burst in human PMN. PMN were exposed to vital *G. intestinalis* trophozoites, PMA, a combination of trophozoites and PMA, TYI-S-33 medium or plain medium (negative control) for 250 min. **(A)** Representative registries of PMN-derived ROS production triggered under different conditions. **(B)** The bar graph shows the area under the curve (AUC) of total ROS production. As expected, PMA-activated PMN resulted in a significant boost of ROS production. In contrast, PMN failed to respond by ROS production to plain parasite exposure (at 1:3 and 1:30 ratios). Interestingly, at 1:30 ratio, *Giardia*-trophozoites significantly inhibited PMA-induced ROS production. *p ≤ 0.05; **p ≤ 0.01; ***p ≤ 0.001; ns, not significant.

Given that ROS are well recognized as anti-parasitic effector molecules and additionally signify key initiators and regulators of the NETotic process, PMN-derived ROS production in response to *G. intestinalis* trophozoites was here assessed by a luminol-based chemiluminescence assay (**Figure 5**). Given that trophozoites were already demonstrated to manipulate and potentially block ROS production, we additionally treated PMN with PMA in absence and presence of *Giardia* trophozoites at 1:3 and 1:30 ratios (**Figure 5**). PMA-stimulated PMN significantly upregulated ROS production (*p* = 0.004) (**Figure 5**). However, the exposure of PMN to *G. intestinalis* trophozoites failed to induce ROS production, irrespective of the MOI, when compared to unstimulated controls (*p* > 0.05) (**Figure 5**). Interestingly, PMA-stimulated PMN in the presence of *Giardia*-trophozoites, showed a dampened ROS production in a dose-dependent manner (1:3 ratio; *p* > 0.05 and 1:30 ratio; *p* = 0.0005) indicating that *G. intestinalis* trophozoites indeed modulate ROS production by PMN (**Figure 5**). To test possible effects of *Giardia*-growth media composition upon PMN-ROS production, PMN were incubated with TYI-S-33 media alone, besides plain PMN and *G. intestinalis* trophozoites as controls (**Figure 5**).

### 3.4 *G. intestinalis* trophozoite-derived ESPs fail to affect PMN metabolic activation and PMN-derived ROS production

**Figure 6:**
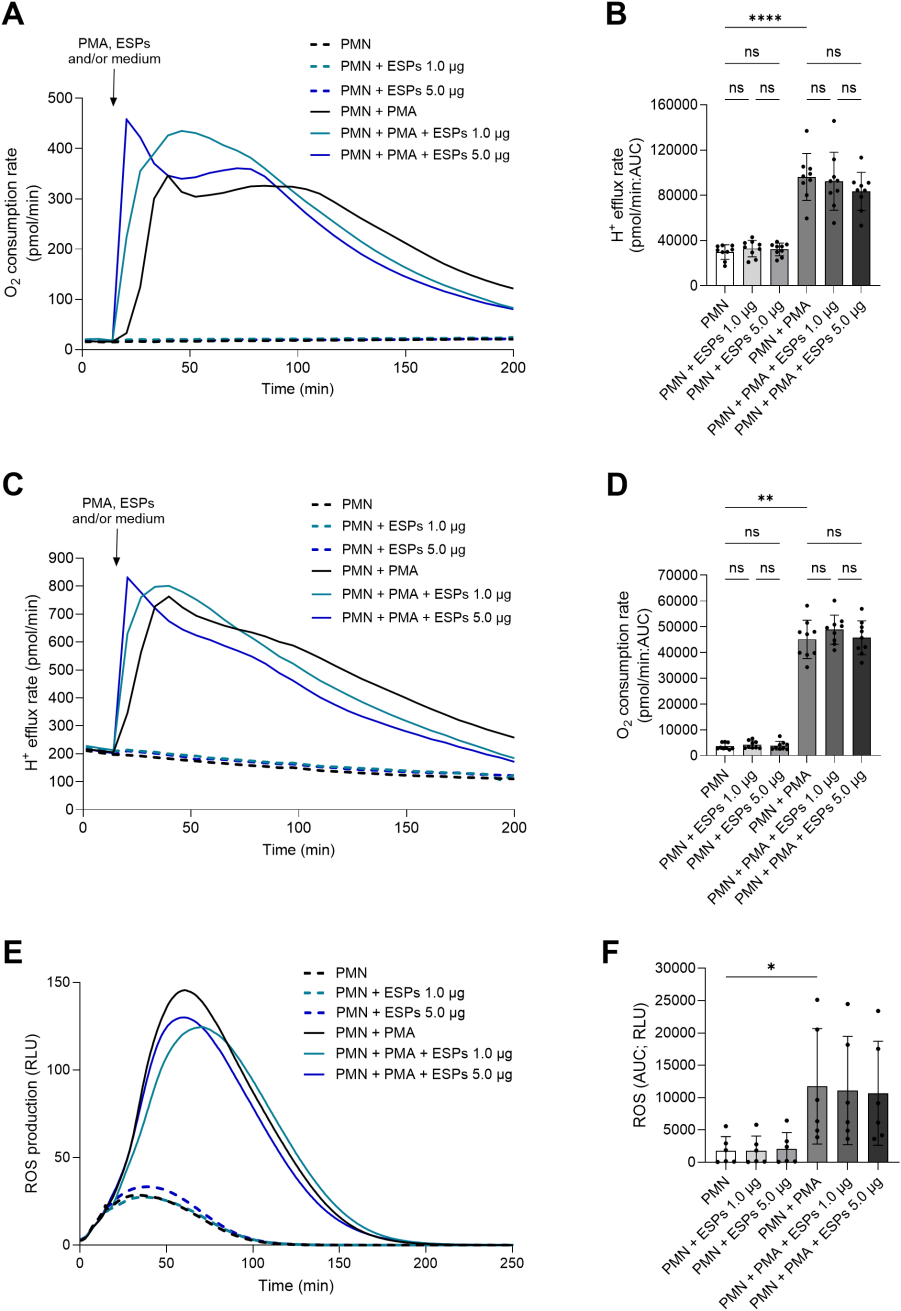
*Giardia intestinalis* trophozoite-derived excretory/secretory products (ESPs) fail to affect bioenergetics and oxidative responses in human PMN. Metabolic responses of PMN exposed to two different concentrations of ESPs were measured via oxygen consumption rate (OCR) and proton efflux rates (PER) followed by ROS production. **(A, C, E)** Representative registries of the bioenergetics status and ROS production of PMN triggered by ESPs exposure. **(B, D, F)** The area under de curve (AUC) was calculated for all registries and plotted as mean ± SD. **(B, D)** Neither OCR nor PER values were significantly affected by exposure ESPs, independently of the concentration, over a period of 200 min. **(F)** No significant effects were observed for PMN exposed to ESPs or in combination with PMA. *p ≤ 0.05; **p ≤ 0.01; ***p ≤ 0.001; ****p ≤ 0.0001; ns, not significant.

Given that the presence of *Giardia* trophozoites dampened neutrophil ROS production, we here studied if trophozoite-derived ESPs may influence PMN responses. Overall, no effects (*p* > 0.05) of *G. intestinalis* ESPs were detectable since they neither induced OCR or PER changes on their own nor affected PMA-driven responses in PMN (**Figures 6A–D**). Moreover, ESPs failed (*p* > 0.05) to induce ROS or to dampen PMA-driven ROS responses in PMN (**Figures 6E, F**). Thereby, suggesting that trophozoite-mediated decrease of PMA-driven ROS production (see 3.3) is independent of *G. intestinalis*-derived-ESPs (**Figures 6E, F**).

### 3.5 Presence of human PMN in *G. intestinalis* cultures dampens trophozoite replication

**Figure 7:**
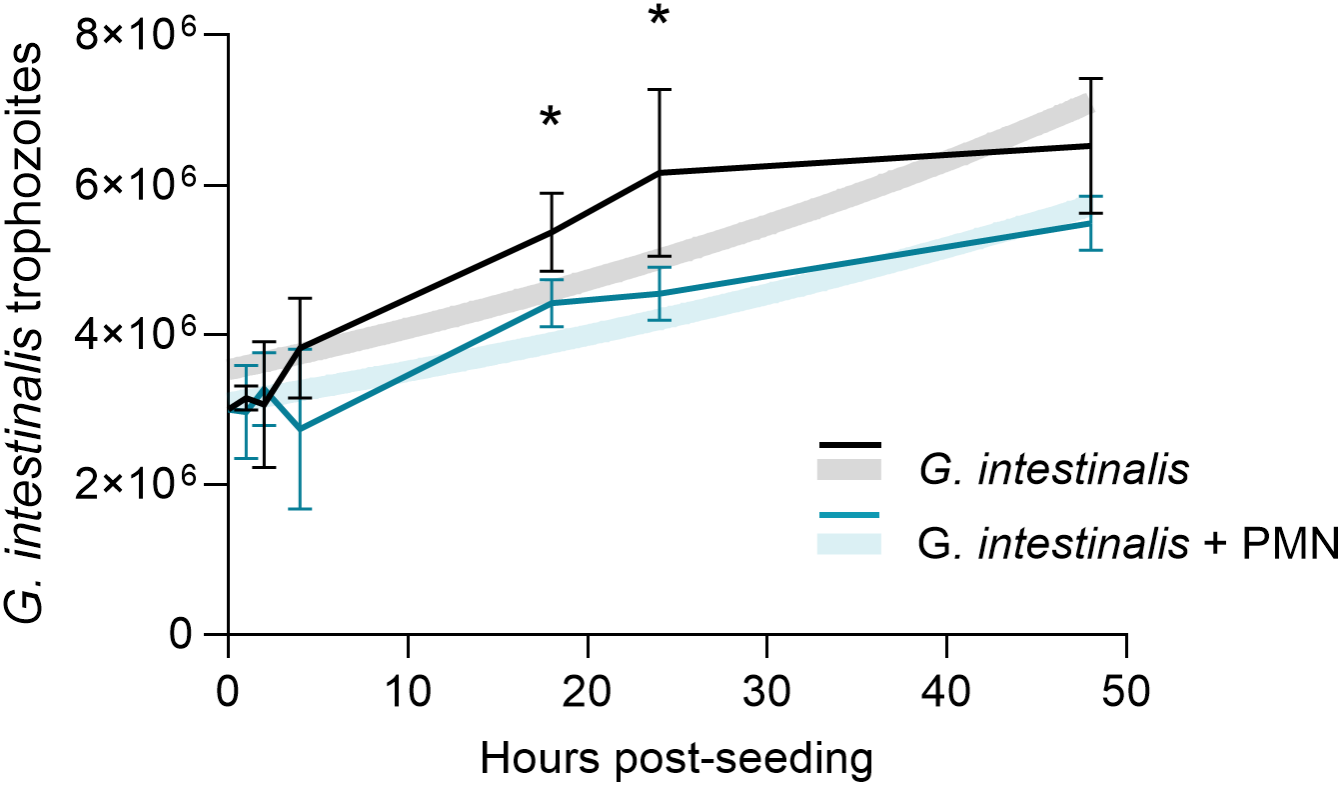
Presence of human PMN in trophozoite cultures dampens *G. intestinalis* growth dynamics. Solid lines represent *G. intestinalis* trophozoite replication in the presence or absence of human PMN (1:3 PMN: *G. intestinalis* ratio). A significant reduction in parasite replication (*p* < 0.05) was observed at 18 and 24 hours post-seeding. Faded background lines represent a comparison of growth curves based on non-linear regression analysis fit to an exponential growth model of the control *G. intestinalis* (faded black line) versus *G. intestinalis* co-cultured with PMN (faded colored line) over a 48-hour period. *p ≤ 0.05.

Given the nature of NETs to entrap microorganisms, possible parasite immobilization may hamper *G. intestinalis* binary fission; therefore, we studied the effects of PMN presence on *G. intestinalis* asexual reproduction dynamics. Interestingly, the presence of PMN led to a significant decrease in *G. intestinalis* duplication since trophozoite numbers at 18 h (*p* = 0.0290) and 24 h (*p* = 0.0387) post-seeding were significantly reduced (**Figure 7**), thereby indicating that PMN-derived anti-parasitic responses indeed affected parasite replication. A non-linear regression analysis fitted to an exponential growth model revealed differences in doubling times between pure *G. intestinalis* cultures (47.67 hours; 95% CI: 36.38 - 69.61) and trophozoite-PMN-cocultures (53.98 hours; 95% CI: 43.62 – 71.17) (**Figure 7**).

## 4 Discussion

In the present study, we show on one hand that human PMN are phenotypically and metabolically activated by *G. intestinalis in vitro*, inducing effector mechanisms such as NET formation and phagocytosis, leading to a moderate dampening of trophozoite replication. On the other hand, ROS production was impaired by *G. intestinalis* by an unknown *Giardia*-driven mechanism. These results highlight that human PMN are indeed being activated, and moreover, that *G. intestinalis* interferes with ROS production, well-known anti-parasitic effector molecules, indicating a potential *G. intestinalis* host immune evasion mechanism. These parasite-immune cell-interactions always have to be considered when transferring *in vitro* data to the *in vivo* system, to better understand outcomes of parasite-induced host-immune response.

Giardiasis is a globally spread intestinal disease of humans, domestic animals and wildlife. *Giardia* trophozoites are extracellular, vegetative stages which sit firmly adhered on the surface of enterocytes, thereby damaging both the microvillous lining and the gut barrier (54–56). Hence, *Giardia*-driven diarrhea was linked to disruption of the micro-villous brush border, villus shortening/atrophy, crypt-hyperplasia, increased epithelial permeability, mucosal inflammation, bacterial overgrowth and intestinal hypermotility (9, 57, 58). Previous findings demonstrated PMN infiltration into *Giardia*-parasitized gut tissue (20). Moreover, *in vitro* studies reported that human neutrophils can interfere with *G. intestinalis* adherence onto IEC (24), which is a prerequisite for gut infection *in vivo*. In the current approach, we mimicked PMN encounter of trophozoite stages *in vitro* and showed by means of SEM and metabolic analyses that PMN are effectively activated upon parasite exposure. Considering neutrophil energy requirements associated with cell activation, we here analyzed real-time changes in the glycolytic and oxidative metabolism of human PMN exposed to vital *G. intestinalis* via Seahorse technology which allows the direct and simultaneous measurement of mitochondrial respiration (i. e. OCR) and glycolytic activity (PER) of cells (59). As expected, exposure of PMN to the chemical stimulant PMA, resulted in a significant increase in OCR- and PER levels, reflecting the metabolic requirements of activated PMN as described elsewhere (60, 61). Moreover, trophozoite exposure resulted in significant upregulation of oxidative and glycolytic PMN responses, thereby reflecting cell activation and mirroring phenotypic PMN changes from resting into activated states, as here illustrated by SEM. Oxidative reactivities of PMN and high O_2_ consumption demands are directly linked to neutrophil respiratory burst (62), i. e. the generation of ROS as anti-parasitic effector molecules. In mammalian PMN, ROS production, mainly through NADPH oxidase (NOX) activity, is considered as an important initial trigger of NETosis (63, 64). Furthermore, we evaluated effects of trophozoite and parasite-derived ESPs exposure to PMN on oxidative burst activities. In our hands, vital *G. intestinalis* did not only fail to upregulate ROS production in human PMN but dampened ROS production driven by PMA. The former finding is in line with Díaz-Godínez et al. (48) and Arbo et al. (65), both reporting on a neutrophil failure to produce ROS in response to (non-opsonized) *G. intestinalis* trophozoites. The latter finding of trophozoite-mediated reduction of PMA-driven ROS production proved to be dose-dependent, since it was almost completely abolished at the 1:30 PMN-to-parasite ratio. This finding raised the question, if the lack of trophozoite-driven neutrophil ROS response is part of *G. intestinalis’* evasion mechanisms. Of note, in order to counteract eventually detrimental ROS, *G. intestinalis* has developed a specialized oxygen detoxification system as sophisticated evasion strategy to thrive in hostile intestinal environments and to escape from ROS-mediated membrane oxidation (66, 67). Hence, this parasite owns several antioxidant enzymes to detoxify O_2_, including NOX, flavodiiron protein, superoxide reductase, flavohemoglobin, and peroxiredoxins (66). Thus, current ROS-related findings may mirror indirect effects of trophozoites, i.e. they may not directly block ROS production but degrade these molecules after release. Given that ROS were not found upregulated in PMN-parasite-co-cultures but even reduced by trophozoite presence in PMA-stimulated PMN, it may reflect the parasite’s potent antioxidant capacity. Taken together, current results indicate that *G. intestinalis*-own antioxidant mechanisms may have a threshold, which may be overcome by a stronger stimulus like PMA stimulation. However, the fact that neither metabolic responses nor ROS production were modulated by trophozoites ESPs, may also argue against the hypothesis of indirect *G. intestinalis*-driven ROS inactivation. Therefore, further *in vivo* or *ex vivo* experimentation is needed to test this hypothesis.

To the best of our knowledge only one previous study examined *G. intestinalis* trophozoite-induced NET formation in humans, but reported on a failure of PMN to extrude NETs in response to these parasite stages (48), thereby contrasting with recent findings on trophozoite-driven NET formation in the bovine and caprine system (47, 68). This discrepancy may be attributed to different experimental settings, donor-dependent variability, parasite strain and/or the selected methodology. However, current data show that exposure of vital *G. intestinalis* trophozoites to human PMN resulted in both metabolic cell activation and low incidence of NET formation. *G. intestinalis*-induced human NETs were confirmed via SEM and by immunofluorescence microscopy by co-localization of granular NE and pan-histone with extruded extracellular DNA-rich structures. Accordingly, NET formation was shown to be induced by several other protozoan parasites like *T. cruzi* (69), *T. brucei brucei* (40), *Leishmania infantum* (70), *T. gondii* (42) and *C. parvum* (71). So far, the molecular mechanisms of vital *Giardia* or parasite-derived ESPs-induced NETosis in PMN remain unknown, however, data from the caprine or bovine system indicate TLR2/4- NOX-, ERK1/2- and p38 MAPK- or PAD4- and P2X1-dependent pathways, respectively (47, 68). In contrast to NETosis-mediated pathogen killing, as described for several bacteria and fungi (72, 73), parasite-induced NET formation primarily seems to result in parasite immobilization (74, 75), thereby either hampering host cell invasion (in case of intracellular protozoa) and parasite stage migration or even to facilitate ongoing site-directed immune reactions. In this context, we showed that the presence of PMN in *G. intestinalis* axenic cultures impaired *G. intestinalis* binary fission leading to a significantly reduced parasite duplication within the first 24 h of culture. Given that PMN failed to generate ROS in response to trophozoites (see above), we here hypothesize that NET-mediated entanglement of parasite stages may have been the driver of replication impairment. To our best knowledge, this is the first report on NET-driven adverse effects on *G. intestinalis* asexual binary fission, which might have implications for human giardiasis outcome *in vivo*.

When considering NET phenotypes, two different kinds of NETs were detected in response to trophozoites, i. e. *spr*NETs and *diff*NETs. Interestingly, *agg*NETs, which were previously reported to be induced by large-sized nematode stages, such as *Haemonchus contortus* (76) and *Dirofilaria immitis* (77), were absent in PMN-trophozoite-co-cultures. Considering the relevance of NET phenotypes *in vivo*, *spr*NETs and *diff*NET release in response to trophozoites may not only be related to host defense but also to the pathogenesis of giardiasis. As such, both *spr*NETs and *diff*NETs are described to own pro-inflammatory properties (38), which might contribute to intestinal mucosal damage by exacerbation, whilst *agg*NETs seem to have anti-inflammatory characteristics (53). Moreover, distinct NETs-components like H2A are reported to induce endothelial- as well as epithelial cell damage (38, 78, 79). Conversely, *agg*NETs are rather associated with chronic phases of inflammation, thereby playing an important role in resolving inflammation *in vivo* (53, 80). The possible role of NET phenotypes on pathogenesis as well as on adaptive immunity against *G. intestinalis* remains poorly studied. Thus, future research focusing on NET phenotypes and their impact on intestinal dendritic cells (DC), tuft cells and gut lymphocytes will significantly contribute to the better understanding of the transition from innate to adaptive immune response.

Besides NET release, SEM analyses also revealed cell activation and active phagocytic activity of single PMN. Even though the size of *G. intestinalis* trophozoites exceeds that of PMN, the partial engulfment of this motile parasitic stage was clearly demonstrated. Interestingly, PMN engage their anti-microbial strategies in accordance with the pathogen’s size and motility (81), which is sensed by mechanoreceptors, such as PIEZO 1 and transient receptor potential vanilloid-type (TRPV) (82, 83). The PMN phagocytic activity observed in the current study agrees with a previous report where attempts of trophozoite phagocytosis by colostral PMN was described (84). So far, it remains unclear whether phagocytosis attempts rather signify an unsuccessful defense mechanism or whether these may contribute to trophozoite replication impairment.

In summary, the current study presents a systematic analysis of early interactions between the enteropathogen *G. intestinalis* and PMN by demonstrating activation and various PMN effector mechanisms. Despite contrasting with a previous report, the current data strongly suggest that *G. intestinalis* trophozoites are recognized by human PMN, thereby enhancing oxidative- and glycolytic levels, NETs and phagocytosis. Our results demonstrate that *G. intestinalis*-triggered NETs release appear to require direct parasite-PMN contact as ESPs failed to induce PMN activation. The molecular mechanisms underlying this process require further elucidation; as well the intracellular signaling pathways involved in NETosis, such as calcium influx, NE, MPO and PAD4 activation. Furthermore, the lack of ROS production by *Giardia*-stimulated PMN suggests a non-canonical PMN response, where NETs are formed without classical activation of the NADPH-oxidase pathway. Finally, *G. intestinalis-*derived antioxidant capacity might evolved as ancestral evolutionary strategy to efficiently evade immunity to infect a wide spectrum of host species. Additionally, NETs-mediated inhibition of parasite’s binary fission might limit intestinal trophozoite colonization thereby contributing to the outcome of human giardiasis.

## Data Availability

The original contributions presented in the study are included in the article/supplementary material. Further inquiries can be directed to the corresponding author.
