# Correction: *Giardia intestinalis* trophozoites activate human PMN and induce NET formation but dampen neutrophil ROS production

**DOI:** 10.3389/fimmu.2026.1829047

**Published:** 2026-03-20

**Authors:** Constanza Salinas-Varas, Taynar L. Bezerra, Lisbeth Rojas-Barón, Luis F. P. Gondim, Florian Wagenlehner, Ulrich Gärtner, Anja Taubert, Carlos Hermosilla, Iván Conejeros

**Affiliations:** 1 Faculty of Veterinary Medicine, Institute of Parasitology, Justus Liebig University Giessen, Giessen, Germany; 2 Department of Anatomy, Pathology and Clinics, School of Veterinary Medicine and Animal Science, Federal University of Bahia, Salvador, Bahia, Brazil; 3 Clinic of Urology, Pediatric Urology and Andrology, Justus Liebig University Giessen, Giessen, Germany; 4 Institute of Anatomy and Cell Biology, Justus Liebig University Giessen, Giessen, Germany

**Keywords:** ESPs, *Giardia intestinalis*, Giardiasis, NET, PMN, ROS

There was a mistake in the caption of **Figure 1** as published. Error: incorrect capitalization and italicization of the scientific name of the parasite. The corrected caption of **Figure 1** appears below.

“*Giardia intestinalis* trophozoites trigger PMN activation, NET formation and phagocytosis in human PMN. (A) PMN-parasite interactions were studied by scanning electron microscopy (SEM). Activated PMN (rough surface) as well as PMN releasing NETs (white arrows) and non-activated PMN (white arrowhead) can be observed in PMN-trophozoite-co-cultures. (B, C) Note released NETs (white arrows) and trophozoites exposing their dorsal surface. (D) Attempt of phagocytosis of *G. intestinalis* trophozoite from a single PMN (white asterisk). The ventral adhesion disc and multiple flagella of a single *G. intestinalis* trophozoite can be observed.”

There was a mistake in the caption of **Figure 2** as published. Error: incorrect capitalization and italicization of the scientific name of the parasite. The corrected caption of **Figure 2** appears below.

“*Giardia intestinalis* trophozoites and *Giardia*-derived excretory/secretory products (ESPs) trigger low incidence NET release in human PMN. (A) PMN were cultured without stimuli (negative control), in the presence of vital *G. intestinalis* trophozoites (MOI 1:3), ESPs, PMA or a combination of both stimuli for 180 min. Immunofluorescence microscopy revealed NET structures by typical extracellularly released structures showing co-localization of decondensated DNA (DAPI, blue), neutrophil elastase (NE, green) and pan-histone (red). (B) Quantification of PMN-NET formation after the exposure to different stimuli. Images are representative of three independent experiments. Scale bars = 20 µm.”

There was a mistake in the caption of **Figure 3** as published. Error: incorrect capitalization and italicization of the scientific name of the parasite. The corrected caption of **Figure 3** appears below.

“*Giardia intestinalis* trophozoites induce the release of different NET phenotypes by human PMN. A spread NET (sprNETs) phenotype is exemplarily illustrated in the upper row. Note the thin and long strings of extracellular DNA (blue) co-localizing with neutrophil elastase (NE; green), and panhistone (red). Diffuse NETs (diffNETs) are exemplarily illustrated in the lower row and consist of roundish structures of decondensated DNA and effector molecules, as demonstrated by the co-localization of DNA (blue), NE (green) and pan-histone (red). Scale bars = 10 µm.”

There was a mistake in the caption of **Figure 4** as published. Error: incorrect capitalization and italicization of the scientific name of the parasite. The corrected caption of **Figure 4** appears below.

“*Giardia intestinalis* trophozoites drive oxidative and glycolytic responses in human PMN. (A, C) The bioenergetic status of parasite-exposed PMN was measured on the level of oxygen consumption (OCR) and proton efflux (PER) rates using a Seahorse XFe96 extracellular flux analyzer over a period of 200 min (B, D). The area under de curve (AUC) was calculated for all registries and plotted as mean ± SD. A significant increase in the oxidative (OCR) and glycolytic (PER) activity of parasite-exposed PMN were observed at a 1:3 ratio and in PMA-activated cells. *p ≤ 0.05; **p ≤ 0.01; ***p ≤ 0.001; ****p ≤ 0.0001; ns, not significant.”

There was a mistake in the caption of **Figure 5** as published. Error: incorrect capitalization and italicization of the scientific name of the parasite. The corrected caption of **Figure 5** appears below.

“*Giardia intestinalis* trophozoites fail to trigger reactive oxygen species (ROS) production and dampen PMA-induced oxidative burst in human PMN. PMN were exposed to vital *G. intestinalis* trophozoites, PMA, a combination of trophozoites and PMA, TYI-S-33 medium or plain medium (negative control) for 250 min. (A) Representative registries of PMN-derived ROS production triggered under different conditions. (B) The bar graph shows the area under the curve (AUC) of total ROS production. As expected, PMA-activated PMN resulted in a significant boost of ROS production. In contrast, PMN failed to respond by ROS production to plain parasite exposure (at 1:3 and 1:30 ratios). Interestingly, at 1:30 ratio, *Giardia*-trophozoites significantly inhibited PMA-induced ROS production. *p ≤ 0.05; **p ≤ 0.01; ***p ≤ 0.001; ns, not significant.”

There was a mistake in the caption of **Figure 6** as published. Error: incorrect capitalization and italicization of the scientific name of the parasite. The corrected caption of **Figure 6** appears below.

“*Giardia intestinalis* trophozoite-derived excretory/secretory products (ESPs) fail to affect bioenergetics and oxidative responses in human PMN. Metabolic responses of PMN exposed to two different concentrations of ESPs were measured via oxygen consumption rate (OCR) and proton efflux rates (PER) followed by ROS production. (A, C, E) Representative registries of the bioenergetics status and ROS production of PMN triggered by ESPs exposure. (B, D, F) The area under de curve (AUC) was calculated for all registries and plotted as mean ± SD. (B, D) Neither OCR nor PER values were significantly affected by exposure ESPs, independently of the concentration, over a period of 200 min. (F) No significant effects were observed for PMN exposed to ESPs or in combination with PMA. *p ≤ 0.05; **p ≤ 0.01; ***p ≤ 0.001; ****p ≤ 0.0001; ns, not significant.”

A correction has been made to the section: Keywords, line 119. Error: Incorrect capitalization and italicization of the scientific name of the parasite. Incorrect capital letter on ESPS. The correct Keywords are:

“KEYWORDS: ESPs, *Giardia intestinalis*, Giardiasis, NET, PMN, ROS”

A correction has been made to the section: **Introduction**, Paragraph Number 1, line 125. Error: Incorrect capitalization and italicization of the scientific name of the parasite. The correct section is:

“*Giardia intestinalis* (syn. *Giardia duodenalis*) is a neglected zoonotic enteric protozoan parasite causing giardiasis, a disease affecting a wide range of hosts, including humans, domestic animals and wildlife (1, 2). This global parasitosis causes more than 300 million human cases of diarrhea annually (3). With a prevalence of up to 30% in developing countries (4), *G. intestinalis* is one of the world’s most prevalent pathogenic protozoa (5). Acute *G. intestinalis* infection in humans induce a wide range of clinical signs including catarrhal diarrhea, cramps, nausea, vomiting, and urticaria (6). Additionally, irritable bowel syndrome (IBS) and chronic fatigue have been reported as sequelae of human giardiasis (6, 7). Although a high percentage of human giardiasis cases may remain asymptomatic and self-limited, life-long consequences, including impaired physical and cognitive development have been reported in *G. intestinalis*-infected young children (5, 8).”

A correction has been made to the section: **Material and methods**, subsection 2.2, title, line 275. Error: incorrect capitalization and italicization of the scientific name of the parasite. The correct section title is:

“2.2 *Giardia intestinalis in vitro* culture”

A correction has been made to the section: **Material and methods**, subsection 2.3, title, line 295. Error: incorrect capitalization and italicization of the scientific name of the parasite. The correct section title is:

“2.3 Isolation of *Giardia intestinalis* excretory/secretory products (ESPs)”

The original version of this article has been updated.

